# Re-Emergence of Bacteriophages and Their Products as Antibacterial Agents: An Overview

**DOI:** 10.3390/ijms26041755

**Published:** 2025-02-19

**Authors:** Vipin Chandra Kalia, Sanjay K. S. Patel, Chunjie Gong, Jung-Kul Lee

**Affiliations:** 1 Department of Chemical Engineering, Konkuk University, 120 Neungdong-ro, Gwangjin-gu, Seoul 05029, Republic of Korea; sanjaykspatel@gmail.com; 2 Cooperative Innovation Center of Industrial Fermentation (Ministry of Education & Hubei Province), Key Laboratory of Fermentation Engineering (Ministry of Education), National “111” Center for Cellular Regulation and Molecular Pharmaceutics, Hubei University of Technology, Wuhan 430068, China; gongcj606@163.com

**Keywords:** biocontrol, biofilm, bacteria, bacteriophage, pathogens, therapy

## Abstract

Microbes possess diverse genetic and metabolic traits that help them withstand adverse conditions. Microbial pathogens cause significant economic losses and around 7.7 million human deaths annually. While antibiotics have historically been a lifesaving treatment, their effectiveness is declining due to antibiotic-resistant strains, prompting the exploration of bacterial predation as an alternative. Bacteriophages (BPhs) have reemerged as antibacterial agents, offering advantages over antibiotics, such as (i) high specificity, (ii) self-replication, and (iii) strong killing capacity. This review explores BPh- and enzyme-based antibacterial strategies for infectious disease treatment, discussing phage–antibiotic synergy, the risks of BPh resistance, and the role of quorum sensing in BPh therapy.

## 1. Introduction

**Figure 1:**
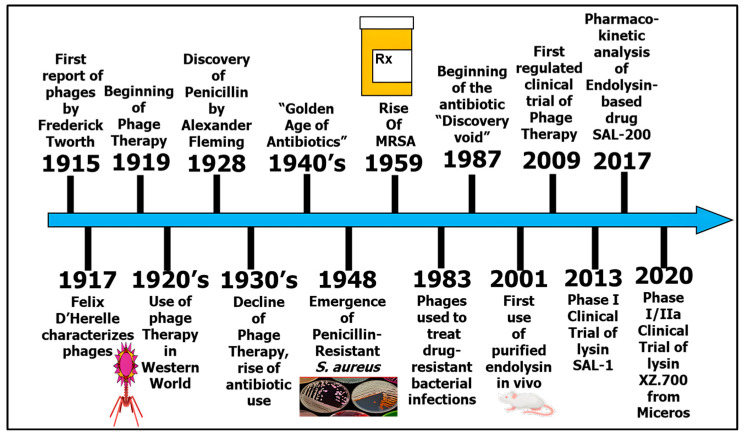
Timeline of phage therapy: Outlined here is the history of phage and endolysin therapy vs. antibiotic therapy, from the discovery of the bacteriophage to the present day. Adapted with permission from [1].

The discovery of bacteriophages (BPhs) in the early 20th century sparked interest in their potential as antibacterial agents [1,2,3]. However, inconsistent results and the rise of antibiotics limited their early use [4]. With the emergence of drug-resistant bacteria and declining antibiotic development [5], interest in BPh therapy (BPhT) has resurged (Figure 1) [1]. BPhs offer key advantages, including specificity, self-replication, rapid adaptation, biofilm clearance, high efficacy, and cost effectiveness [2,3,6]. Despite their known potential [7,8], only a few former USSR countries have approved BPh-based treatments for human use [9]. The global demand for new BPhs remains high due to evolving pathogens and regulatory challenges [10].

Bacterial infections significantly contribute to morbidity and cause 7.7 (5.7–10.2) million deaths annually [11,12]. Notably, 65–80% of chronic infections involve biofilm-forming bacteria, making treatment more challenging. Key pathogens include Gram-negative bacteria (e.g., *Acinetobacter*, *Escherichia*, *Klebsiella*, and *Pseudomonas*), responsible for respiratory and urinary infections, and Gram-positive bacteria (e.g., *Enterococcus*, *Staphylococcus*, and *Streptococcus*), which cause soft tissue infections [13,14,15,16]. Biofilm removal strategies focus on preventing bacterial adhesion, inducing detachment, and disrupting biofilm regulation through quorum sensing (QS) inhibitors (QSIs) and matrix degradation [17,18].

**Figure 2:**
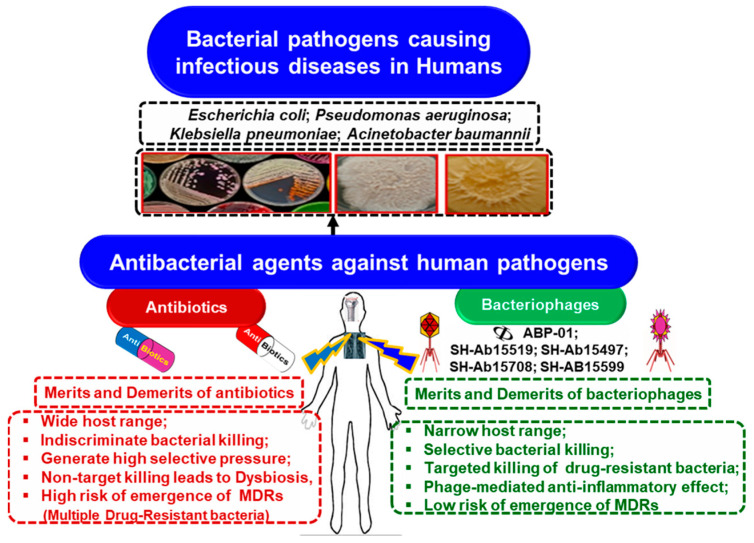
Bacterial pathogenic infections in humans and the comparative merits and demerits of phage therapy versus antibiotics.

The urgent need for alternative antibacterial approaches has intensified. While biofilm inhibition methods are well documented [19], recent reviews rarely discuss BPhs as tools against infectious pathogens [20]. This review examines BPh-based antibacterial strategies (Figure 2), including phage–antibiotic synergy, the risks of BPh resistance, and the role of QS in BPhT.

A bibliographic analysis was conducted, reviewing research articles and review papers indexed in Scopus, PubMed, and Google Scholar. The search utilized keywords including biocontrol, biofilm, bacteria, bacteriophage, phage enzymes, pathogens, therapy, phage cocktails, antibiotics, antibacterial agents, infectious diseases, phage resistance, quorum sensing, horizontal gene transfer, and endolysins. Approximately 800 studies, primarily from the last decade, were critically evaluated to synthesize the key findings and advancements in the field.

## 2. Bacteriophages as Antibacterial Agents

Bacteriophages (BPhs) infect bacteria through lytic and lysogenic cycles [21]. In the lytic cycle, phages hijack bacterial machinery, replicate, and lyse the host cell, making them ideal for therapeutic use. In contrast, the lysogenic cycle involves phage DNA integrating into the bacterial genome, remaining dormant. While lysogenic phages pose risks due to horizontal gene transfer (HGT) of resistance or toxin genes, they hold potential for bacterial genetic engineering. Strictly lytic phages are essential for clinical applications to ensure safety and effectiveness. Prophages can also provide immunity against superinfections [21]. Lytic BPhs interact with biofilms at different stages [22,23] using three main antibacterial strategies: (i) whole phages, (ii) bacteriophage-derived enzymes (e.g., exopolysaccharide polymerases and peptidoglycan hydrolases), and (iii) phage–antibiotic combinations.

Early research on monophage therapy showed promising results (Table 1). A few examples are that (i) the BPh EF-P29 prevented bacteremia and alleviated gut dysbiosis in a vancomycin-resistant *Enterococcus faecium* (VREF) murine model [24], (ii) the BPh SHEF2 eradicated *Enterococcus faecalis* biofilms and improved survival in zebrafish infected with *E. faecalis* OS16 [25], and (iii) lytic phages significantly reduced bacterial loads, with phage ph0034 decreasing counts by 7.5 log CFU/mL and ph0031 by 5.1 log CFU/mL within 24 h [26]. Monophage therapy also showed efficacy against *Staphylococcus aureus* infections: (i) In a mouse *S. aureus* lung infection model, lytic BPhs achieved 100% survival within 24 h, compared to 62% with clindamycin alone and 75% with combination therapy [27]. (ii) The BPh SLPW reduced inflammation in methicillin-resistant *S. aureus* (MRSA)-infected mice [28]. (iii) Georgian *Staphylococcus* BPhs have successfully treated diabetic foot ulcers and *S. aureus* infections [29].

Phage cocktails enhance treatment consistency by preventing biofilm formation and reducing BPh-resistant mutants [30] (Table 1). Successful applications include the following: (i) reducing epithelial cell damage and apoptosis in enterohemorrhagic *Escherichia coli* EHEC O157 infection by 57.3% [31], (ii) reducing *E. coli* ST131-H30R gut colonization [32], (iii) inhibiting biofilm formation in levofloxacin-resistant *Pseudomonas aeruginosa* [33], (iv) treating *Acinetobacter baumannii* wound infections and preventing necrosis [34], (v) rescuing mice from acute and chronic bloodstream infections [35], (vi) enhancing efficacy against *A. baumannii* with specific BPs [36]; (vii) reducing inflammation in VREF-induced septic peritonitis [37]; (viii) phages targeting *E. faecalis* and *E. coli* improved gut microbiota in murine colitis models [38], and (ix) the *Kayvirus* phages SAM1 and SAM2 in the Fersisi cocktail effectively infected MRSA strains, although host gene expression changes require further study [39]. Despite their advantages, phage therapy faces challenges, including phage resistance, HGT risks, dysbiosis, cross-resistance, and high production costs [40].

**Table 1:** Bacteriophages as biocontrol agents against bacterial pathogens.

Bacterial Pathogen	Bacteriophage Infection Site	Antimicrobial Activity	Application/Target	References
*Bacteriophages*				
*Escherichia coli* strain DPC6051	ɸAPCEc01, ɸAPCEc02, and ɸAPCEc03	Complete inhibition of bacterial growth and biofilm formation	Human health	[30]
*E. coli* O157:H7	Phage (CA933P) and probiotics (lactobacilli and yeasts)	Cell detachment: 1.2 log CFU; reduced apoptotic cell count by 57.3%	Controlling pathogenic infection and reducing epithelial cell damage	[31]
*Pseudomonas aeruginosa* strain DS38	Cocktail (φKMV, φPA2, φPaer4, and φE2005-24-39)	Inhibited biofilm formation and reduced the preformed biofilms	Improving host ranges and using defined cocktails to reduce UTIs	[33]
*Acinetobacter baumannii* AB5075	Cocktail of AB-Army1 and AB-Navy1-4 (5 × 10^9^ PFU)	AB-Army1 targeted the capsulated pathogen, and the rest (AB-Navy1-4) lysed the pathogen, preventing the spread of infection and necrosis in a mice wound	Eradicate wound infection in humans	[34]
*A. baumannii* AB9 (MDR)	Cocktail of vB_AbaS_D0 and vB_AbaP_D2 (10^9^ PFU/mL)	Improved the therapeutic efficacy and reduced the frequency of phage-resistant bacteria in murine bacteremia (intraperitoneally)	Improved therapeutic efficacy; reduced phage-resistant bacteria	[36]
*Staphylococcus aureus*	vB_SauM_phiIPLA-RODI; vB_SepM_phiIPLA-C1C	2-log reduction in cell adherence and removed 5 log units of planktonic cells in 8 h	Therapeutic application	[22]
*S. aureus* (MDR)	Phage (10^8^ PFU/mL)	Survival rate of 100% in mice (intravenous)	Public health: anti-drug resistance	[27]
*Enterococcus faecalis* (VR)	Lytic phage EF-P29 (4 × 10^5^ PFU, intraperitoneally)	Protected all mice against bacteremia (2 × 10^9^ PFU/mouse)	For treating life-threatening nosocomial infections and avoiding gut microbiota imbalance	[24]
*E. faecalis* (VR)	Lytic (EFDG1 and EFLK1)	Rescued mice from severe septic peritonitis (100%) in a mouse model	For treating life-threatening nosocomial infections and avoiding gut microbiota imbalance	[35]
*E. faecalis* EF54	SHEF2	Eradicated biofilms on polystyrene surfaces and on tooth root	Broad-range biocontrol agent against antibiotic-resistant infections	[25]
*E. faecalis* EF3964	vB_EfaS_PHB08 (10^5^ CFU/cm^2^)	Reduced bacteria (10^5^ CFU/cm^2^) on a polystyrene MTP and lettuce as a vegetable model	For treating wounds and UTIs in animals and humans	[41]
*Bacteriophagal enzymes*				
*S. aureus* V329	vB_SepiS-phiIPLA7 (EPS depolymerase Dpo7; 0.15 μM)	Exopolysaccharide matrix degradation (30%); removal of biofilm-attached cells: 90%	Controls hospital-acquired infections and bacteremia	[22]
*S. aureus*	Muralytic enzymes P128 from phage K (≥12.5 μg/mL)	Removal of biofilms up to 95.5%	Infectious conditions (chronic rhinosinusitis)	[42]
	Lysin CF-301 (≤0.25 μg/mL)	Eradication of biofilm within 1 h and bacterial killing within 6 h on catheters, surgical mesh, glass, and polystyrene surfaces	Treating staphylococcal infections	[43]
*E. faecalis* (MDR and VR strains)	Endolysin LysEFm5 (from IME-EFm5; 16 to128 mg/mL)	Lysis of 19/23 pathogenic isolates in actively growing cells	Highly specific against MDR Gram-positive pathogens	[44]
*E. faecalis* EF3964	Endolysin lys08 (from vB_EfaS_PHB08; 5 µg)	Eradicated the biofilm on a polystyrene MTP	For treating wounds and UTIs in animals and humans	[41]

CFU, colony-forming units; MDR, multidrug resistant; PFU, plaque-forming units; VR, vancomycin resistant; UTI, urinary tract infection.

## 3. Bacteriophage Enzymes as Antibacterial Agents

Phage enzymes target bacterial surface structures, such as lipopolysaccharides, exopolysaccharides (EPSs), and capsular polysaccharides [45]. Examples include (i) *Pseudomonas putida* phage 815 tail spike proteins binding and degrading bacterial capsules [46,47], (ii) phage polymerases reducing biofilms by 37% within 24 h at an MOI of 10^6^ [48], (iii) *S. epidermidis* phage phiPLA7 depolymerase degrading 30% of the EPSs in *S. aureus* biofilms [49], and (iv) phage ϕAB6 tail proteins degrading *A. baumannii* EPSs, demonstrating therapeutic potential [50]. Peptidoglycan-degrading enzymes offer high specificity and efficacy, independent of bacterial resistance mechanisms [51]. These include glycosidases, lysozymes, amidases, and endopeptidases [52]. A few examples are as follows: (i) murein hydrolase P128 and lysostaphin from BPh K disrupted 95.5% of *S. aureus* sinus biofilms at ≥12.5 μg/mL [42], (ii) endolysin LysH5, CHAPk peptidase, and lysin ClyH effectively degraded *S. aureus* biofilms [53,54,55], (iii) endolysin Lys68 combined with a membrane permeabilizer reduced *Salmonella Typhimurium* by 1 log unit [56], (iv) the BPh lysin CF-301 eradicated 90% of *S. aureus* biofilms within one hour at ≤0.25 μg/mL [43], (v) LysEFm5 from the BPh IME-EFm5 showed activity against vancomycin-resistant *E. faecium* without requiring Zn ions [44], and (vi) BPh vB_EfaS_PHB08 endolysin reduced *E. faecalis* by 10^5^ CFU, showing promise for wound and UTI treatments [41].

Engineered chimeric BPhs are being developed to comply with regulatory requirements, easing the approval process for phage cocktails. Key strategies to enhance BPh efficacy include (i) avoiding identical receptor targets, (ii) using diverse phage species, and (iii) introducing novel BPhs to counteract resistance [9,57]. A low resistance rate to phage lytic proteins has been observed, supporting their potential as future therapeutic agents [58,59].

## 4. Complementing Phages with Antibiotics

BPhs enhance antibiotic efficacy through synergistic effects, improving outcomes using the BPh Henu2 [60], against MRSA [61], and in dental infections [62]. Examples include the following: (i) a *Burkholderia cepacia* phage with low-dose meropenem improved *Galleria mellonella* survival [63], (ii) phage–ciprofloxacin reduced *P. aeruginosa* load by 10,000-fold in endocarditis-infected rats [64], (iii) ciprofloxacin post-BPh infection eliminated *E. coli* more effectively than antibiotics or phages alone [65], (iv) the lytic *S. aureus* phage Sb-1 with oxacillin exhibited synergy [61], (v) daptomycin combined with *E. faecium* phage cocktail showed limited efficacy [66], (vi) phages 6 and 45 with gentamicin reduced *P. aeruginosa* by 3 logs in 12 h [60], (vii) phage MRM57 with cefotaxime showed synergy against *Citrobacter amalonaticus* [67], and (viii) flucloxacillin enhanced *S. aureus* biofilm suppression in rats (2.15 log CFU/g reduction) [68]. Although PAS (phage–antibiotic synergy) is generally beneficial, some antagonistic interactions occur [69,70].

PAS is regulated through multiple mechanisms. Certain antibiotics stimulate phage replication, increasing progeny release. Quinolones and β-lactams induce bacterial elongation, enhancing susceptibility to phage lysis enzymes in Yersinia enterocolitica and *E. coli* [71]. Ceftriaxone combined with a Siphoviridae phage inhibited *P. aeruginosa* cell wall synthesis by triggering *sulA* gene activation, leading to filamentation and increased phage assembly [72]. Some antibiotics enhance the plaque and burst size, e.g., the T4 phage burst size increased 5-log at low cefotaxime levels, reducing its latent period [73]. PAS reduces resistant mutants, as seen with ciprofloxacin/daptomycin and S. aureus phage Sb-1 [64,74]. Phage–antibiotic interactions enhance antibiotic susceptibility by depleting lysogens [75], re-sensitizing pathogens [76], and reducing resistance [77].

### 4.1. PAS Against Bacterial Biofilms

Phage–antibiotic combinations effectively eradicate biofilms: (i) a T4 phage with cefotaxime lowered the minimum eradication concentration for *E. coli* biofilms [73], (ii) T4 and PB-1 phages with antibiotics eliminated 60–99% of biofilm biomass and reduced phage-resistant cells by 39–99% in *E. coli* and *P. aeruginosa* infections [78,79], (iii) ciprofloxacin-resistant *E. coli* biofilms were cleared using the phage ɸWL-3 and fosfomycin [80], (iv) the phage vB PmiS-TH with ampicillin significantly reduced *P. mirabilis* biofilms, with greater effects at higher antibiotic doses and phage MOI [81], and (v) phage cocktails showed effectiveness against MDR *A. baumannii* biofilms in a murine model [82]. These findings underscore PAS’s potential in eradicating bacterial biofilms and combating antibiotic resistance.

### 4.2. In Animal Models

Phage–antibiotic synergy (PAS) has shown efficacy in animal infection models:

- In rat osteomyelitis, the *P. aeruginosa* PAT14 and *S. aureus* Sb-1 phages combined with antibiotics significantly reduced biofilm formation [83].
- In a mouse post-arthroplasty model, the *S. aureus* MR-5 phage (10^9^ PFU/mL) and linezolid (5%) lowered the bacterial load by day 10 [84].
- For *K. pneumoniae* pneumonia, the phage P-KP2 with gentamicin increased survival to 70% in mice [85].
- In neutropenic mice with *P. aeruginosa* lung infections, the PEV20 phage (10^6^ PFU/mg) and ciprofloxacin (0.33 mg/mg) led to a 5.9-log bacterial reduction [86].

However, PAS was less effective in some cases: (i) in a rat MRSA model, linezolid monotherapy reduced mortality by 38%, while phage treatment had variable survival rates [87]; (ii) a *S. aureus* phage cocktail with daptomycin in MRSA pneumonia showed limited efficacy (50–55% survival) [88]. PAS outcomes vary based on the antimicrobial type, dosage, and treatment timing.

### 4.3. In Humans

#### 4.3.1. Case Reports

BPhT has been applied to treat diverse bacterial infections (Table 2):

- A 2-year-old child with *P. aeruginosa* sepsis and congenital heart disease improved with a phage–antibiotic combination, although their symptoms recurred post-therapy [89].
- A post-aortic aneurysm repair patient with ciprofloxacin-resistant *P. aeruginosa* was treated with the phage OMKO1 (10^7^ PFU/mL) and ceftazidime, eradicating the pathogen within four weeks, with no recurrence for two years [90,91].
- In seven cardiothoracic patients (ages 13–66) with MDR infections, phage therapy was effective and well tolerated [92].
- In a 61-year-old man with *E. cloacae* peritonitis and MDR *P. aeruginosa* septicemia, BFC1 improved his renal function, although he later died of *K. pneumoniae* sepsis [93].
- A renal transplant patient’s recurrent *K. pneumoniae* UTI was successfully treated with a personalized phage [94].
- A 63-year-old man with a UTI from XDR *K. pneumoniae* was treated with a lytic BPh cocktail, remaining infection-free at six months [95].
- Another patient with MDR *K. pneumoniae* gut colonization received an oral and intra-rectal lytic BPh, showing no adverse effects [96].
- In cystic fibrosis (CF) cases, the following was found:
  ○ A 17-year-old infected with *B. cepacia* and *Achromobacter xylosoxidans* showed improved lung function (FEV1 from 1.83L to 3.33L) after inhaled phage therapy [97].
  ○ A 26-year-old patient with MDR *P. aeruginosa* recovered following eight-week phage cocktail (AB-PA01) treatment and antibiotics, with no pneumonia recurrence after 100 days [98].
  ○ Engineered phage therapy stabilized post-lung transplant CF patients with *Mycobacterium abscessus* infections [99].
- A necrotizing pancreatitis patient with *A. baumannii* pseudocyst infection recovered following IV phage treatment after resistance to initial cocktails [100].
- Ventilator-associated pneumonia and empyema patients tolerated a four-phage cocktail well [101].
- Phage Sb-1 effectively treated *S. aureus* toe ulcers in nine patients over seven weeks [29].
- BPhT, used alongside systemic antibiotics, successfully treated biofilm infections from ventricular assist devices, enabling heart transplants in MSSA and *P. aeruginosa* cases, although serum-neutralizing activity and BPh titers require further study [102].
- In a study with 62 infected patients and 30 healthy volunteers, the sera’s anti-phage activity did not influence outcomes, although 40–55% of patients showed positive responses [103].
- In critically ill patients with *S. aureus* infections treated with a BPh (AB-SA01), the inflammatory markers decreased over 90 days. Future studies will use a 12 h dose of 10^9^ PFU/mL to assess the BPhT’s efficacy [104].
- In an 80-year-old woman with prosthetic joint infections (*P. aeruginosa* and *S. aureus*), phage therapy yielded lasting positive results for 18 months [105].
- For severe musculoskeletal infections, BPh cocktails were administered intraoperatively every eight hours for ten days alongside antibiotics, with no recurrence or severe side effects for up to sixteen months [106].
- Personalized BPhs as adjuvants to antibiotics successfully treated *P. aeruginosa* infections in periprosthetic joints, allowing the patient to regain mobility within ten months [107].
- A 42-year-old patient with bone infections from XDR *A. baumannii* and MDR *K. pneumoniae* avoided amputation with phage therapy [108].

**Table 2:** Potential applications of bacteriophages as against infectious diseases: case reports and clinical trials.

Pathogenic Bacteria	Bacteriophage (BPh)	Disease and Patient Details	Mode of Administration	Outcomes	References
*Pseudomonas aeruginosa* (MDR, Colistin-only sensitive)	Cocktail BFC1 (2 BPhs)	Septicemia (acute kidney injury, 61-year-old male)	Intravenous (IV) (10^9^ PFU/mL, every 6 h for 10 days)	Recovery from septicemia achieved after 10 days of treatment	[93]
*P. aeruginosa*	OMKO1	Aortic graft infection (76-year-old male)	IV (10^7^ PFU/mL) + ceftazidime	Pathogen eradication within four weeks, with no recurrence reported after two years	[91]
*P. aeruginosa* (MDR)	Cocktail (2 BPhs)	Bacteremia (2-year-old male child)	IV (3.5 × 10^5^ PFU/mL, every 6 h for 3 days) + antibiotics	Symptoms returned after stopping therapy	[89]
*P. aeruginosa* (XDR)	Cocktail	Bone metastasis (60-year-old male with lung cancer)	Applied on bone in the cavity (10^8^ PFU/mL)	Positive results lasting 18 months	[105]
*P. aeruginosa* and *Staphylococcus aureus* (methicillin-susceptible)	Cocktail of *P. aeruginosa* and *S. aureus* BPhs	Prosthetic joint infection (80-year-old obese woman with type 2 diabetes mellitus and chronic kidney injury)	Intraoperative injection in the joint cavity	Positive results lasting 18 months	[105]
*P. aeruginosa* (MDR, Colistin-only sensitive)	Cocktail AB-PA01 (4 lytic BPhs)	Pneumonia in cystic fibrosis (CF, 26-year-old female)	IV (4 × 10^9^ PFU/mL, every 6 h for 8 weeks)	Recovery with no recurrence of pneumonia after 100 days	[98]
*P. aeruginosa*	Cocktail AB-PA01 (4 lytic BPhs)	Ventilator-associated pneumonia and empyema (77-year-old female)	IV (10^9^ PFU/mL, every 12 h for 7 days) and nebulized	No adverse effects	[101]
*P. aeruginosa PsA (MDR)*	Lytic BPhs (BAP-5phi1, PFU +MTAE-8 phi1, and PFU + MTAE-8 phi3) + antibiotics (meropenem, ceftezidine/avibactam + aztreonam)	HeartMate II driveline infection; recurrent bacteremia	IV (1.5 × 10^6^ PFU + 2.2 × 10^9^ PFU + 2.1 × 10^7^ PFU in 1 mL every 8 h for 6 weeks)	Lytic BPhs allowed the patients to proceed with heart transplantation successfully	[102]
*P. aeruginosa* and *S. aureus*	Cocktail of *P. aeruginosa* (PNM and 14-1) and *S. aureus* (ISP) BPhs	Chronic osteomyelitis	Intraoperatively (10^7^ PFU/mL, every 8 h for 7–10 days)	No recurrence or severe side effects up to 16 months	[106]
*P. aeruginosa* (MDR)	*P. aeruginosa* BPh	Knee periprosthetic joint infection and chronic osteomyelitis (80-year-old woman with metabolic syndrome, diabetes, etc.)	IV (10^8^ PFU/mL, every 8 h for 5 days)	Patient achieved notable recovery and mobility after 10 months	[107]
*Acinetobacter baumannii* TP1 (MDR)	Cocktail ϕPC followed by cocktail ϕ IV (AB-Navy1, AB-Navy4, AB-Navy71, and ABNavy97)	Diabetes (68-year-old patient)	IV (10^9^ PFU/mL for 36 h followed by increasing frequency for 2 days for period of 11 weeks) + antibiotics	Rapid recovery over 12 weeks	[100]
*A. baumannii Ab*KT722 (XDR) and *Klebsiella pneumoniae KP*KT1 (MDR)	Cocktail of ɸAbKT21phi3 and ɸKpKT21phi1	Bone infection (42-year-old male)	IV (5 × 10^7^ PFU/mL, every 8 h for 5 days)	BPhs and antibiotics resulted in avoiding amputation	[108]
*K. pneumoniae*	*K. pneumoniae* BPh	UTI–epididymitis (58-year-old male)	Administered orally and into the bladder through catheter; treatment continued for 12 weeks along with antibiotics	Eradicated a recurrent UTI, showing high efficacy one year later	[94]
*K. pneumoniae* (XDR) CX10301	Cocktail of lytic BPhs (Kp152, Kp154, Kp155, Kp164, Kp6377, and HD001; 5 × 10^8^ PFU/mL of each BPh) + sulfamethoxazole–trimethoprim	Recurrent UTI (63-year-old male)	Intravesical phage administration and oral antibiotics; bladder irrigation every 12 h for 5 days	Remained infection-free at six months	[95]
*K. pneumoniae* strain ST307 (MDR and carbapenemase producing)	Lytic BPh	Recurrent obstructive nephrolithiasis and UTIs (57-year-old female)	Administered orally and via intra-rectal modes over a 3-week cycle	No adverse effects	[96]
*S. aureus*	Staphylococcal BPh Sb-1	Diabetic foot ulcers (44–92 years old, 6 males)	Topical (0.1–0.5 mL of 10^7^–10^8^ PFU/mL)	Effectively treated poorly vascularized toe ulcers in nine patients over seven weeks	[29]
*S. aureus (MSSA)*	Cocktail AB-SA01 (3 lytic BPhs: Sa87, Sa83, and Sa36) + antibiotics (cefazolin + minocycline)	HeartMate II driveline; sternal osteomyelitis; recurrent bacteremia	IV (3 × 10^9^ PFU in 1 mL every 12 h for 4 weeks)	Allowed the patients to proceed with heart transplantation successfully	[101]
*Mycobacterium abscessus* subsp. *massiliense* GD01	Cocktail (3 lytic BPhs: Muddy, ZoeJ Δ*45*, and BPh *33*ΔHTH-HRM10)	CF (15-year-old female)	IV (10^9^ PFU in 5 mL, every 12 h for 32 weeks)	Stabilized post-lung transplantation in cystic fibrosis patients	[99]
Antibiotic-resistant bacteria	Cocktails (MS-1 and OPMS-1): *S. aureus*, *E. faecalis*, and other BPhs	62 patients, infections of the genitourinary tract, prostatitis, bone, respiratory tract, skin, or soft tissue	Intra-rectal or local (10^6^–10^9^ PFU/mL, every 8–12 h for 12 weeks or more)	40–55% of patients showed positive responses	[103]

MDR, multidrug resistant; MSSA, methicillin-sensitive *Staphylococcus aureus*; PFU, plaque-forming units; UTI, urinary tract infection; XDR, extensively drug resistant.

- A craniectomy patient with *A. baumannii* infection received a lytic phage cocktail, but rapid phage clearance led to treatment withdrawal; the patient died on day 20 [109].
- Lung transplant patients with *Burkholderia dolosa* and *P. aeruginosa* (MDR) were treated using phages. The *P. aeruginosa* patients recovered, but those with *B. dolosa* infections relapsed, leading to mortality [110].
- A critically ill patient with respiratory infection caused by *A. baumannii* improved after 35 days of treatment with the phage AbW4878Ø1 (1 × 10^9^ PFU/mL) and broad-spectrum antibiotics [111].

While phage therapy shows promise, factors such as serum-neutralizing activity, dosing, and pathogen susceptibility require further study.

#### 4.3.2. Clinical Trials

- The PhagoBurn trial tested a 12-phage cocktail (PP1131) for *P. aeruginosa*-infected burn wounds. The low-dose treatment (1 × 10^6^ PFU/mL) took 144 h to meet the primary endpoint, compared to 47 h for silver sulfadiazine. The trial was terminated early due to poor efficacy, likely due to inadequate phage dosage (200–2000 PFU vs. the typical 2–3 × 10^7^ PFU/mL) [112].
- A trial on *E. coli*-associated diarrhea in children was abandoned due to ineffectiveness. A follow-up study on 79 children tested two oral phage cocktails (M: 1.4 × 10^9^ PFU/mL, T: 3.6 × 10^8^ PFU/mL) over four days, showing no adverse effects but comparable efficacy to the placebo. Likely, the issues included gastric degradation and poor intestinal delivery, highlighting the need for improved formulations for *E. coli* in Asia [113,114].
- A trial on MRSA bacteremia and endocarditis evaluated exebacase, an anti-staphylococcal lysin. A single IV dose with standard antibiotics led to superior clinical responses and good tolerability in MRSA patients compared to antibiotics alone [115,116].
- Of the numerous phase I/II BPhT studies, only two have reached phase III: (i) nebulized pyoBPh complex [117] and (ii) post-transurethral prostate resection intervention for UTI patients [118]. Some recent trials have shown contradictory outcomes [119].
- A phase I trial on chronic rhinosinusitis tested the intranasal phage cocktail AB-SA01 (up to 3 × 10^9^ PFU/mL) over two weeks in a tertiary center, demonstrating safety and efficacy. These findings suggest phages as potential antibacterial agents for this condition [120].

## 5. Limitations

BPhs are proposed as an alternative to antibiotics but require efficacy validation before clinical trials. Key challenges include phage resistance, gut microbial dysbiosis, HGT, and cross-resistance.

### 5.1. High Specificity and Microbial Documentation

BPhs target specific bacterial strains, minimizing microbiota disruption but limiting broad therapeutic application [50,51]. Effective treatment requires precise pathogen identification using advanced diagnostics like MALDI-TOF, whole-genome sequencing, or culture-based methods [121,122]. Pre-treatment phage testing (e.g., plaque assays and biofilm disruption studies) ensures infectivity and lytic activity against clinical isolates. Customized phage cocktails may be needed for polymicrobial infections or rapidly evolving pathogens [123].

BPh infections can alter bacterial metabolism, as seen in *S. aureus* biofilms treated with phiIPLA-RODI, which showed reduced peptidoglycan biosynthesis, potentially affecting virulence and resistance [124,125]. The gut microbiome, containing ~10^12^ viruses (mostly BPhs), facilitates HGT, potentially spreading antibiotic resistance and virulence factors [126,127]. While temperate phages contribute to HGT, their role in human health remains unclear [128,129]. Gut microbiome alterations have been linked to obesity, diabetes, immune disorders, and cardiovascular diseases [130,131,132,133].

BPh safety is evaluated by its impact on gut microbiota and host tissues. While BPhs can eliminate antibiotic-resistant bacteria, endotoxin release may trigger immune responses. No severe adverse effects have been reported [134]. The phage vB_KpnM_GF effectively controlled a *K. pneumoniae* outbreak, suggesting potential for decolonizing multidrug-resistant pathogens [135].

### 5.2. Dose, Interval, and Administration Optimization

Challenges in BPh therapy (BPhT) include formulation, propagation, and stability. There is insufficient knowledge on maintaining phage viability at room temperature, affecting their shelf life and usability. Delivery methods like inhalation (for respiratory infections) and oral administration (for gastrointestinal infections) require formulations that protect phages from degradation in physiological conditions [136,137].

To optimize BPhT, the focus has shifted to purified phage enzymes like endolysins and cell wall hydrolases, which offer enhanced bacterial targeting and reduced gene transfer risks [48]. Regulatory clarity on BPhT practices is essential for clinical application [138,139].

### 5.3. Regulatory and Safety Considerations

Phages, being “living” entities, complicate regulatory approval by agencies like the FDA and EMA [140]. While phage therapy is used in parts of Europe, standardized guidelines are lacking [141,142]. Pharmacokinetic data on endolysins are limited, and while BPh-related side effects (e.g., skin allergies) are rare, further study is needed [143].

Lytic BPhs are preferred over temperate ones to prevent virulence and resistance gene transfer. Phage cocktails with EPS polymerases improve biofilm targeting [144]. The immune system’s response, particularly anti-phage antibodies, can affect therapy outcomes, as seen in phage MS-1 treatment, where 23% of patients with pre-existing antibodies showed reduced efficacy [145,146].

Endolysins degrade bacterial cell walls via glycosidases, amidases, or endopeptidases, leading to osmotic lysis. Unlike BPhs, they degrade quickly, reducing gene transfer risks. Studies on PaI and CpI-1 lysins showed no immune-related side effects, stable catalytic activity, and no toxicity [147,148]. Recombinant phage proteins, such as thermostable endolysins, hold promise but require further safety and immunogenicity studies [149].

### 5.4. Emergence of BPh-Resistant Bacteria

Like antibiotics, BPhs can drive bacterial resistance. Viral fitness factors may enhance bacterial survival, promoting resistance to both antibiotics and phages [150,151]. In *P. aeruginosa*, mutations in *hmgA* and *galU* affected O-antigen expression, preventing phage adsorption [152]. In *L. monocytogenes*, a loss of cell wall teichoic acid residues due to phage A511 infection reduced bacterial fitness [153,154]. However, resistant mutants could aid in developing live attenuated vaccines [155].

CRISPR/Cas-mediated immunity also enables bacteria to recognize and clear phages, although this reduces bacterial fitness at low CRISPR array scales [156,157,158,159]. Biofilms hinder phage adsorption by creating physical barriers, while competitive inhibitors may block phage receptors [160]. *S. aureus* infection by phiIPLA-RODI induced biofilm formation through altered peptidoglycan biosynthesis, conferring resistance. Thus, lytic phages that release plasmid DNA should be avoided in BPhT [124,161].

## 6. Emerging Role of Bacterial QS in Bacteriophage Infection

Quorum sensing (QS), a key bacterial communication system, regulates biofilm formation, phage adsorption, the lytic–lysogenic cycle, genetic exchange, and phage–host coevolution [162,163]. QS exerts dual effects on phage infections, either inhibiting or promoting them. For example, in *Vibrio anguillarum*, QS inhibits phage infection by reducing phage receptor expression. A *∆vanT* mutant exhibits increased *OmpK* receptor expression at low cell densities, promoting biofilm formation and phage resistance. Conversely, at high cell densities, the *∆vanO* mutant suppresses prophage induction and enhances proteolysis, preventing biofilm formation [164,165].

QS activity is quantified using (1) chemical assays (e.g., HPLC, GC-MS, fluorescence/absorbance assays, and bioassays), (2) gene expression analysis (e.g., reporter gene systems, qRT-PCR, and RNA-Seq), (3) phenotypic assays (e.g., biofilm quantification via crystal violet staining and motility assays), and (4) mathematical modeling to determine QS dynamics [166].

QS molecules such as N-acyl-homoserine lactone (AHL), CAI-1, and AI-2 modulate phage resistance by downregulating lipopolysaccharide (LPS) O-antigens, reducing *Vibrio cholerae* susceptibility to phages [167]. Phage infection can also activate QS genes, enhancing biofilm formation in *P. aeruginosa* and *E. coli* [168]. Indole, a QS signal, inhibits phage infection in *P. aeruginosa* by downregulating the genes essential for type IV pilus (T4P) assembly, reducing phage adsorption [169]. Similarly, in *Shewanella baltica*, QS decreases phage adsorption via LuxR-mediated LPS receptor downregulation [170].

Phage–QS interactions remain underexplored. Phages influence QS-regulated virulence throughout infection [171]. In *P. aeruginosa*, QS upregulates *bci*, impacting motility, biofilm formation, and pyocyanin production, critical for infections in CF patients [172]. Additionally, *las-QS* in *P. aeruginosa* PAO1 enhances phage infection by upregulating *galU*, increasing phage-mediated killing efficiency [173]. In *V. cholerae*, phages regulate QS-controlled biofilm genes [174] (Table 3).

Cyclic-di-GMP (c-di-GMP) modulates biofilm dynamics, and the phage PB1 disrupts biofilms by producing anti-c-di-GMP peptides [175]. QS inhibitors (QSIs) prevent biofilm formation, increasing bacterial susceptibility to phages. However, when QS promotes phage infection, QSIs may reduce phage therapy efficacy. A sequential strategy using QSIs and phages is advisable when QS does not enhance phage infection, facilitating bacterial eradication at lower antibiotic doses [17].

Future research should critically evaluate QS-mediated biofilm formation, a major barrier to effective phage therapy. Strategies focusing on bacterial adhesion prevention, biofilm matrix degradation, and QS-targeted interventions require extensive investigation to enhance BPhT efficacy.

## 7. Conclusions

Bacteriophage therapy (BPhT) emerged nearly a century ago but was overshadowed by antibiotics. The rise of multidrug-resistant pathogens and stagnation in antibiotic development have renewed interest in alternatives like QSIs. Although promising against various infections (e.g., respiratory, urinary tract, burn wounds, and endocarditis), BPhT lacks regulatory approval as a standard treatment. Studies highlight the potential of PAS in reducing antibiotic use and limiting resistance, but further research is needed to clarify synergistic and antagonistic effects. Addressing these challenges could integrate BPhT into clinical practice as a vital tool in the post-antibiotic era.

A critical research focus should include QS and QSI strategies targeting bacterial adhesion, polysaccharide production, and biofilm degradation. Combining QS-targeted interventions with phage therapy can significantly improve treatment efficacy and clinical outcomes.

## Figures and Tables

**Table 3:** Bacterial quorum-sensing-mediated regulation of bacteriophage infection.

Bacteria	Quorum Sensing System	Bacteriophage	Effect of Phage Infection	Reference
*Pseudomonas aeruginosa* ATCC 10798	*sdiA*, *luxS*, *lasI,* and *lasR*	PEB1 and PEB2	Upregulates QS genes (*lasI* and *lasR* of *las* QS system); enhanced biofilm formation, which negatively affect BPhT	[168]
*P. aeruginosa* ATCC 27853	*lasI*, *pslA*, *lasB,* and *phzH*	vB_PaeM_USP_1, vB_PaeM_USP_2, vB_PaeM_USP_3, and vB_PaeM_USP_18	Phage infection regulates the expression of QS-mediated virulence-related genes and the outcome of BPhT	[171]
*P. aeruginosa*	Type IV pilus (T4P)	vB_Pae_S1 and vB_Pae_TR	Indole, a potential QS signal, prevents phage infection by downregulating the expressions of genes *pilA*, *pilB*, and *pilQ*, which are essential for type IV pilus (T4P) assembly, reducing phage adsorption	[170]
*Vibrio anguillarum* PF430-3	*vanT*	KVP40	Downregulation of QS-mediated OmpK anti-phage defense strategy through enhanced biofilm formation; QS blocked phage infection by reducing phage receptors	[165]
*V. anguillarum* 90-11-287	*vanT* and *vanO*	ϕH20-like prophage	QS represses prophage induction; high proteolytic activity represses biofilm formation, adversely affecting BPhT	[165]
*Vibrio cholerae* C6706*lacZ*	AHLs (CAI-1 and AI-2)	JSF35	QS signal molecules (N-acyl-homoserine lactone (AHL), CAI-1, and AI-2) downregulate the phage receptor—lipopolysaccharide O-antigen—improving bacterial resistance to phages	[167]
*V. cholerae* C6706	VqmA	VP882	Phage influences the expression of QS-regulated genes for biofilm formation	[174]
*Escherichia coli* ATCC 15692	*sdiA*, *luxS*, *lasI,* and *lasR*	PEB1 and PEB2	Phage interactions upregulate genes regulating QS secretion (*sdiA* and *luxS* of *lux* QS system); higher biofilm matrix, which negatively affect BPhT	[168]
*Shewamella baltica*	LuxR	e vB_Sb_QDWS	QS regulated resistance to phage infection by decreasing levels of lipopolysaccharide-mediated phage adsorption via downregulation of genes *galU* and *tkt*, which are critical for phage receptor synthesis	[170]
*P. aeruginosa* CC274	*lasR*, *rhlR*, *qscR*, and *pqsR*	PHAGE_ Pseudo_ phi297_ NC_ 016762-like phage	QS increases the expression of the *bci* gene, influencing motility, biofilm formation, and pyocyanin production, which supports the phage infection capacity and helps in managing bacterial infections in CF patients	[172]
*P. aeruginosa* ATCC 15692	*las*	vB_Pae_QDWS	Las-QS in *P. aeruginosa PAO1* promotes phage infection by upregulating galU, enhancing the phage infection and phage-mediated killing efficiency	[173]

AHL: QS signal acyl-homoserine lactone; QS signal CAI-1 (*S*-3-hydroxytridecan-4-one); AI-2: autoinducers-2; bci: bacteriophage control infection. las system consists of a transcriptional activator, *lasR*, and *lasI* (gene) encodes for LasB: elastase; *lasI*: autoinducer synthase; *luxS*: *S*-ribosylhomocysteinase; *phzH*: transamidase; *pslA*: polysaccharide synthesis; *sdiA*: suppressor of cell division inhibitor; *vanT*: transcription factor for serine racemase; *vanO*: σ^54^-dependent response regulator; *vqmR*: regulatory RNA; *galU*: UTP-α-d-glucose-1-phosphate uridylyltransferase; *tkt*: transketolase, EC 2.2.1.1.

## Data Availability

No new data were created or analyzed in this study. Data sharing is not applicable to this article.

## Abbreviations

BPh	Bacteriophage
BPhT	Bacteriophage therapy
QS	Quorum sensing
QSI	Quorum sensing inhibitor
VREF	Vancomycin-resistant *Enterococcus faecium*
PFU	Plaque-forming units
CFU	Colony-forming units
MRSA	Methicillin-resistant *Staphylococcus aureus*
HGT	Horizontal gene transfer
EPS	Exopolysaccharides
MOI	Multiplicity of infection
MIC	Minimal inhibitory concentration
PAS	Phage–antibiotic synergy
UTI	Urinary tract infection
CF	Cystic fibrosis
CRISPR	Clustered regularly interspaced short palindromic repeats
